# Gene Expression Driven by a Strong Viral Promoter in MVA Increases Vaccination Efficiency by Enhancing Antibody Responses and Unmasking CD8^+^ T Cell Epitope

**DOI:** 10.3390/vaccines2030581

**Published:** 2014-07-22

**Authors:** Pablo D. Becker, Miriam Nörder, Sebastian Weissmann, Ronny Ljapoci, Volker Erfle, Ingo Drexler, Carlos A. Guzmán

**Affiliations:** 1 Department of Vaccinology and Applied Microbiology, Helmholtz Centre for Infection Research, D-38124 Braunschweig, Germany; E-Mails: pablo.becker@kcl.ac.uk (P.D.B.); noerder@clinical-trial-center.de (M.N.); sebastian.weissmann@helmholtz-hzi.de (S.W.); 2 Institute of Virology, Technische Universität München, D-81675 Munich, Germany; E-Mails: ronny.ljapoci@med.uni-duesseldorf.de (R.L.); volker.erfle@googlemail.com (V.E.); ingo.drexler@med.uni-duesseldorf.de (I.D.); 3 Laboratory for Molecular Virology, Institute for Virology, Düsseldorf University Hospital, Heinrich-Heine-University, D-40225 Düsseldorf, Germany; 4 Clinical Cooperation Groups “Antigen Specific Immunotherapy” and “Immune-Monitoring” Helmholtz Centre Munich, D-81675 Munich, Germany

**Keywords:** modified vaccinia virus Ankara, vaccine vector, promoter, delivery system, vaccine

## Abstract

Viral vectors are promising tools for vaccination strategies and immunotherapies. However, CD8^+^ T cell responses against pathogen-derived epitopes are usually limited to dominant epitopes and antibody responses to recombinant encoded antigens (Ags) are mostly weak. We have previously demonstrated that the timing of viral Ag expression in infected professional Ag-presenting cells strongly shapes the epitope immunodominance hierarchy. T cells recognizing determinants derived from late viral proteins have a clear disadvantage to proliferate during secondary responses. In this work we evaluate the effect of overexpressing the recombinant Ag using the modified vaccinia virus early/late promoter H5 (mPH5). Although the Ag-expression from the natural promoter 7.5 (P7.5) and the mPH5 seemed similar, detailed analysis showed that mPH5 not only induces higher expression levels than P7.5 during early phase of infection, but also Ag turnover is enhanced. The strong overexpression during the early phase leads to broader CD8 T cell responses, while preserving the priming efficiency of stable Ags. Moreover, the increase in Ag-secretion favors the induction of strong antibody responses. Our findings provide the rationale to develop new strategies for fine-tuning the responses elicited by recombinant modified vaccinia virus Ankara by using selected promoters to improve the performance of this viral vector.

## 1. Introduction

Several candidate vaccines against infectious diseases and cancer, based on poxviral vectors, are under clinical evaluation. One of these vectors is the modified vaccinia virus Ankara (MVA), which was attenuated by performing more than 500 passages in chicken embryo fibroblast cultures [1,2]. During its attenuation, MVA lost ~15% of its parental genome, including genes that regulate viral host range and evasion of host immune response. Dissemination within the host is precluded in most species, including humans, due to the extremely impaired ability to replicate in mammalian and, particularly, in human cells [3,4,5]. This results from a block in virion morphogenesis at a late stage of infection, thus, replication deficiency has no apparent effect on viral or recombinant gene expression [3,4,5].

MVA also showed an excellent safety record when administered during the smallpox eradication campaign in approximately 150,000 individuals, including many persons at risk for the conventional smallpox vaccines [6,7,8]. Recombinant MVA (rMVA) expressing immunogens from a variety of infectious agents or tumor-associated antigens (Ags) have been successfully tested in clinical trials [9,10,11,12,13,14,15,16,17,18,19]. One of the most important characteristics of MVA is its ability to target dendritic cells (DCs) *in vivo*. This is a prerequisite for the induction of an adaptive immune response, not only by MVA but also by other poxviruses [20,21,22,23,24,25,26,27]. Thus, MVA is a promising viral vector for vaccines and immunotherapies. However, CD8^+^ T cell responses triggered against multiple pathogen-derived epitopes expressed by rMVA are characterized by defined immunodominance hierarchy patterns, which, in turn, limit the potential usefulness and efficacy of this viral vector. We have demonstrated that the timing of viral Ag expression in infected professional Ag-presenting cells (APCs) strongly shapes the epitopes immunodominance hierarchy [28]. However, the timing of Ag expression is not the only factor, since MVA normally cannot replicate in mammalian hosts. Hence, the amount of Ag produced is also limited and the usage of enhanced promoters may be advantageous. Thus, we explored whether an increased amount of recombinant target Ag could be an adequate strategy to enhance the immunogenicity of antigenic determinants delivered by rMVA. To this end, we investigated the effect resulting from driving Ag expression by the strong genetically modified vaccinia virus early/late promoter mPH5 [29]. Although the overall expression levels did not significantly differ from those obtained with the conventional promoter P7.5 (P7.5), the early promoter element of mPH5 drives a stronger expression in the early phase of infection. Interestingly, this difference in expression patterns has a direct impact on the CD8^+^ T cell responses by unmasking epitopes which otherwise would have been rarely detected. Furthermore, the increase of secreted Ag leads to the induction of enhanced antibody (Ab) responses, which reach levels similar to those achieved by the administration of soluble Ag with non-rMVA (nrMVA) as adjuvant [30]. Thus, the results presented in this study suggest that the use of rMVA encoding an Ag for which expression is driven by different promoters is an effective approach to selectively potentiate and modulate immune responses to vaccination.

## 2. Experimental

### 2.1. Ag, Peptides and Media

Ovalbumin (OVA, Sigma, St Louis, MO, USA) was used as Ag for *in vitro* and *in vivo* studies. The MHC class I (H-2k^b^)-restricted OVA dominant (aa 257-264, SIINFEKL), subdominants (aa 11–18, CFDVFKEL and aa 55–62, KVVRFDKL) peptides [31,32,33], and the MHC class II (I-A^b^)-restricted OVA peptide (aa 323–339, ISQAVHAAHAEINEAGR) [34] were synthesized and HPLC purified (>99% purity) at the Helmholtz Centre for Infection Research (HZI, Braunschweig, Germany).

All cell cultures were performed with complete medium: RPMI 1640 (Gibco, Carlsbad, CA, USA) supplemented with 10% heat inactivated FBS, South American origin (Greiner Bio-One GmbH, Frickenhausen, Germany), 100 U/mL of penicillin (Gibco), 50 µg/mL streptomycin (Gibco), 1 mM l-glutamine (Gibco) and 50 µg/mL gentamycin (Sigma).

### 2.2. Mice

Female C57BL/6 mice six to eight weeks old were purchased from HarlanWinkelmann GmbH (Borchen, Germany). Mice were kept under specific pathogen-free conditions in individual ventilated cages with food and water *ad libitum* OT-I mice expressing the OVA_257–264_/K^b^-specific T cell receptor (TCR) and OT-II mice expressing the OVA_323–339_/A^b^ specific TCR on C57BL/6 background have been described elsewhere [35,36,37]. Mice were propagated and maintained in the animal facility of the HZI. Mice were housed and handled in accordance with good animal practice as defined by the Federation for Laboratory Animal Science Associations and the national animal welfare body Gesellschaft für Versuchstierkunde/Society of Laboratory Animals and experiments were performed in compliance with the German animal protection law (TierSchG BGBl. S. 1105; 25.05.1998). All animal experiments were approved by the local authorities, permission number: 509.42502/07-04.01, Bezirksregierung Braunschweig.

### 2.3. Plasmid Construction

In order to generate the MVA vector plasmids pIIIΔHR-mPH5-OVA and pIIIΔHR-P7.5-OVA, a 1.3 kb DNA-fragment containing the entire coding sequence of the OVA gene was excised with *Eco*R I and *Xba* I from plasmid pcOVA (a generous gift from H. Wagner, Institute of Immunology, Munich, Germany), modified by Klenow enzyme, and cloned into a unique *Pme*I restriction site of pIIIΔHR-mPH5 or PIIIΔHR-P7.5 [38,39]. In these vector transfer plasmids the expression of OVA is under the control of the modified vaccinia virus early/late promoter mPH5 [29,40] or the natural vaccinia virus early/late promoter P7.5 [41], respectively.

### 2.4. Viruses

MVA(II_new_), the vaccinia virus MVA cloned isolate optimized for host range selection, was routinely propagated and titrated by endpoint dilution in chicken embryo fibroblasts (CEFs) to obtain a 50% tissue culture infectious dose (TCID_50_) [38,39]. For *in vitro* and *in vivo* assays, MVA wild type was purified by ultracentrifugation through a 36% sucrose cushion. Vaccine preparations were reconstituted in 1 mM Tris pH 7.4, 120 mM NaCl saline buffer.

### 2.5. Generation of Recombinant Viruses

Recombinant OVA expressing viruses (MVA-OVA) were obtained by homologous recombination using the transfer plasmids pIIIΔHR-mPH5-OVA or pIIIΔHR-P7.5-OVA, respectively, followed by transient K1L-based host-range selection as described previously [38]. Briefly, CEFs infected with MVA(II_new_) at a multiplicity of 0.01 TCID_50_ per cell were transfected with transfer plasmid DNA, harvested and processed as described previously [38]. MVA expressing the OVA gene and transiently co-expressing host range-coding sequences (K1L) were isolated by consecutive rounds of plaque purification in RK13 cells. MVA expressing only the OVA gene were isolated by additional rounds of plaque purification on CEF cells. The recombinant viruses MVA-OVA P7.5 and MVA-OVA mPH5 were subsequently amplified in CEF monolayers and viral DNA genomes were analyzed by PCR. High titre stocks of purified rMVA were prepared by ultracentrifugation through a 36% sucrose cushion. Vaccine preparations were reconstituted in 1 mM Tris pH 7.4, 120 mM NaCl saline buffer. To ensure that comparable titres were used for the different MVA constructs, titrations were performed by qPCR and cell-based assays.

### 2.6. Western Blot Analysis

Expression of OVA was confirmed by Western blot analysis of cell lysates from infected A375 cells. For some experiments, infections were performed in the presence of AraC (Sigma-Aldrich, St. Louis, MO, USA) at 40 µg/mL to restrict the MVA gene expression to the early phase of the viral replication cycle. Epoxomicin (Sigma) was used at 0.5 µM, which not only inhibits degradation of proteins produced in cells after viral infection but also blocks expression of intermediate and late genes. Total protein content for each sample was determined by DC Protein Assay (Biorad, Hercules, CA, USA). After loading 80 µg total protein per sample, proteins were resolved by electrophoresis through SDS 12% polyacrylamide gels and electroblotted onto nitrocellulose for 1 h in a buffer containing 25 mM Tris, 192 mM glycine, 0.037% SDS, and 20% methanol, pH 8.3. Blotting efficacy was controlled by staining with Ponceau S solution. Blots were then blocked overnight on ice in a Tris-buffered saline blocking buffer containing 1% BSA, 0.1% Tween-20, and 0.02% NaN_3_, and further incubated for 2 h at room temperature with an OVA-specific Ab (rabbit polyclonal to OVA, Ab1221, Abcam, Cambridge, England) diluted 10,000-fold in blocking buffer. Blots were washed with 0.1% Tween-20 in Tris-buffered saline and incubated for 1 h at room temperature with HRP-conjugated goat polyclonal anti-rabbit IgG (Dianova GmbH, Hamburg, Deutschland) diluted 15,000-fold. The blots were then washed, incubated with chemiluminescent substrate (Lumi-Light; Roche, Basel, Switzerland), and exposed to a photographic film (BioMax; Kodak, Rochester, NY, USA).

### 2.7. Bone Marrow Derived DC Preparation, in Vitro Stimulation and Injection

BM-DCs were prepared from the femur and tibia of C57BL/6 mice using rGM-CSF (BD Pharmingen, San Diego, CA, USA) as previously described [42,43]. Briefly, legs were harvested and all muscle tissues were removed from the femur and tibia. Epiphyses were cut off and bones were flushed out with medium using a 26G-needle. After incubation at 37 °C for 2 h of BM cells in tissue culture dishes, non-adherent cells were collected and re-plated in 6-well plates at 1 × 10^6^ cells/mL in complete medium supplemented with 10 ng/mL rGM-CSF, and further incubated for 6–7 days at 37 °C with 5% CO_2_. On day 3 of culture, half of the medium was replaced by fresh medium supplemented with rGM-CSF. On day 6 of culture, cells were counted, mixed and divided into 6-well plates to have 5 × 10^6^ cells/well. Only cultures that showed more than 70% of CD11c^+^ cells at day 6 were used for experiments. DCs were infected with different concentrations of MVA (MOIs of 0.05, 0.5, and 5) for 6 h. Then, DCs were washed 3 times with PBS in order to remove unbound MVA and further incubated for 16 h. Thereafter, the expression of DCs surface markers was analyzed by flow cytometry or functional assays were performed.

### 2.8. Flow Cytometric Analysis

The DC stimulation was evaluated by surface marker expression by flow cytometry. Briefly, cells were incubated with mouse Fc block (BD Pharmingen) at 4 °C for 15 min. Then, cells were stained with fluorescence-labelled Abs diluted in fluorescence-activated cell sorting (FACS) buffer (PBS-BSA 1%). FITC-labelled mAb against mouse CD40 (3/23), CD80 (16-10A1), CD86 (GL1), H2-K^b^ (AF6-88.5) and I-A^b^ (A6-120.1), as well as PE- or PE-Cy7-labeled mAb against CD11c (HL3 and N418, respectively) were purchased from BD Pharmingen and eBioscience (San Diego, CA, USA). After 30 min of incubation at 4 °C in the dark, cells were washed twice with FACS buffer. Then, cells were resuspended, transferred to FACS tubes and kept on ice in the dark until analysis, which was performed on a FACSCalibur or a FACS Canto (BD Bioscience) using BD cell Quest^TM^Pro or FACS Diva software and gating on CD11c^+^ cells. The viability of the stimulated DCs was analyzed using Vybrant^TM^ Apoptosis Assay Kit #2 (Molecular Probes, Leiden, The Netherlands), according to the manufacturer’s instructions. Apoptosis and death of infected DCs were assessed by staining with Annexin V labelled with AlexaFluor488 and 7-AAD, respectively. Analysis of apoptotic/dead cells was done gating on CD11c^+^ cells, using FlowJo software and to the data presented as histograms was normalized by expressing them as % of the Max.

### 2.9. Proliferation Assay

The Ag processing and presentation by DCs was examined by the proliferation of Ag-specific naïve T cells after co-culture with the differentially pre-treated DCs. This test was performed using naïve T cells from OT-I and OT-II transgenic mice. Spleens from OT-I or OT-II mice were aseptically removed and pooled. Organs were mechanically disaggregated in complete RPMI medium by gently pressing them through a 100 µm mesh using a syringe plunger. The cell suspension was centrifuged and the pellet was resuspended in ACK buffer to lyse erythrocytes. CD4^+^ and CD8^+^ T cells were isolated using the CD4^+^ or CD8^+^ T cell isolation kit (Miltenyi Biotec GmbH, Bergisch Gladbach, Germany) from OT-II and OT-I mice, respectively. For the [3H]-thymidine incorporation assay, pre-treated DCs were added in quadruplicates at different T cells:DCs ratios (ranging from 6.25 to 50) to flat-bottom 96-well culture plates. As positive controls, DCs stimulated with either the corresponding OVA peptides (0.1 μg/mL) or the OVA protein (10 μg/mL) were used. T cells were labelled with 1 µM carboxyfluorescein diacetate succinimidyl ester (CFSE, Molecular Probes). Labelling was stopped by adding FBS. After one wash in complete medium, T cells were adjusted to 1 × 10^6^ cells/ml and 100 µL of the cell suspension was co-cultured with pre-treated DCs in 96-well plates. Cell suspensions were seeded in round-bottom 96-well cell culture plates (TPP, Trasadingen, Switzerland). As positive control, T cells were stimulated with concanavalin A, as negative control T cells were cultured without stimulus in complete medium. Plates were incubated for 5 days at 37 °C with 5% CO_2_. On day 5, CD4^+^ and CD8^+^ T cells were stained with PE-Cy7 labelled CD4 (RM4-5, eBioscience, San Diego, CA, USA) and allophycocyanin labelled CD8 (53-6.7, eBioscience) Abs, respectively. Proliferation was measured by flow cytometry using a LSR-II FACS machine (BD Bioscience, San Jose, CA, USA) interfaced to the FACS Diva Software (BD Bioscience).

### 2.10. Staining for Intracellular Cytokines

Restimulated cells were stained for surface markers, fixated by 2% paraformaldehyde and subsequently incubated in permeabilization buffer, PBS containing 0.5% saponin (AppliChem, Frankfurt, Germany) and 0.5% BSA for 1 h. Abs for intracellular staining were diluted in permeabilization buffer and added to the cells for 30 min. Cells were washed with permeabilization buffer and PBS and analyzed by flow cytometry using a LSR-II FACS machine (BD Bioscience).

### 2.11. In Vitro Ag Presentation Assay

BMDCs were generated as described before and infected with MVAs at MOIs of 1 and 10 for 6 h or loaded with OVA protein or peptide AA323–339 for 6 h. Then, BMDCs were washed 3 times with PBS in order to remove unbound MVA or Ag. Naïve CD4^+^ T cells were isolated from splenocytes of OT-II mice by FACS. Cells were stained for CD4 (PE-Cy7, RM4-5, eBioscience), CD44 (APC, IM7, eBioscience), CD25 (PE, PC61, BD Bioscience), and CD62L (FITC, Mel-14, BD Bioscience), sorted for CD4^+^CD25^−^CD44^lo^CD62L^hi^ using an Aria-II FACS machine (BD Bioscience) and labelled with CFSE. In a 96-well plate 10^4^ pre-treated BMDCs were co-cultured with 2 × 10^4^ or 10^5^ naïve CD4^+^ T cells. At day 5, cells were restimulated with ionomycin (1 µg/mL, Sigma) and phorbol 12-myristate 13-acetate (PMA, 0.01 µg/mL, Sigma) for 4 h. For the last 2 h brefeldin A was added (5 µg/mL, Sigma). Cells were stained for CD4 (PE-Cy7, RM4-5, eBioscience), viability (fixable live/dead, near-IR, Life Technologies, Eugene, OR, USA) and intracellularly for IL-4 (APC, 11B11, eBioscience) and IFN-γ (PB, XMG1.2, eBioscience). Cytokine responses were analyzed by gating on viable, divided CD4^+^ cells with FlowJo software [44].

### 2.12. Immunization Studies

Groups of six C57BL/6 mice were immunized by i.m. route into the biceps femoris using a 25G needle on day 1 and boosted on day 28 (100 µL/dose/mouse) with 1 × 10^8^ PFU of MVA per dose. Negative controls received PBS and nrMVA alone. Serum samples were collected from blood of the tail vein 1 day before each immunization and 1 week after the last immunization when the mice were sacrificed by CO_2_ inhalation. Sera were stored at −20 °C prior to determination of specific Abs.

### 2.13. Detection of Ag Specific Abs

OVA-specific Abs were determined by ELISA, as previously described [45]. For details see Supplementary Information.

### 2.14. Detection of IFN-γ-Producing Cells by ELISPOT

The number of IFN-γ-secreting cells in the spleen of immunized mice was determined by ELISPOT assay, according to the manufacturer’s instructions (BD Bioscience).

### 2.15. Detection of IFN-γ-Producing CD4^+^ T Cells by Flow Cytometry

The frequency of cytokine producing CD4^+^ T cells in the spleen of immunized mice was determined by restimulating splenocytes with OVA (40 µg/mL) for 24 h. For the last 6 h brefeldin A (5 µg/mL) was added. The restimulated splenocytes were stained for CD3 (V500, 500A2, BD Bioscience), CD4 (PE-Cy7, RM4-5, eBioscience), CD8 (FITC, 53-6.7, BD Bioscience), viability (fixable live/dead, near-IR, Invitrogen, Carlsbad, CA, USA) and intracellularly for IFN-γ (PB, XMG1.2, eBioscience). Cytokine responses were analyzed by gating on viable CD3^+^CD4^+^CD8^−^ cells with the FlowJo software [44].

### 2.16. Statistical Analysis

Statistical analysis was performed using the Graphpad Prism software [46]. The significance of the differences observed between three or more groups was determined using the one-way ANOVA followed by the Turkey Kramer post test. Differences were considered significant at *p* < 0.05.

## 3. Results

### 3.1. The Use of Different Promoters Results in Changes in the Profile and Turnover of Proteins Expressed by rMVA in Infected Cells

**Figure 1:**
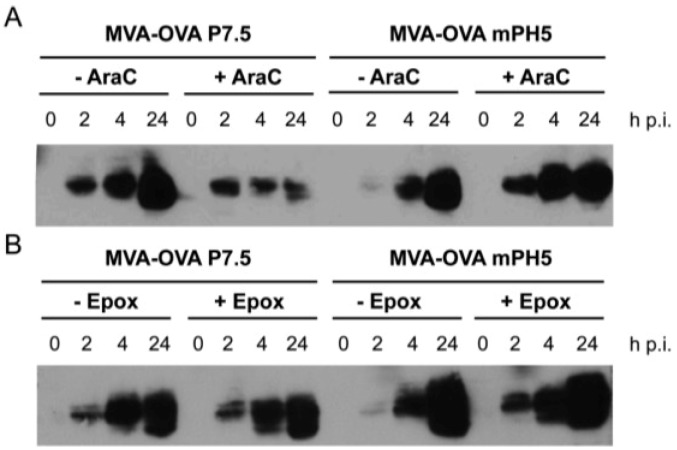
Expression under control of mPH5 results in degradation of gene products at early time points. Human A375 cells were infected with either MVA-OVA P7.5 or MVA-OVA mPH5 at MOI 10. Cells were harvested at the indicated time points (h p.i., hours post infection) and cell lysates were prepared. (**A**) Cytosine β-d-arabinofuranoside (AraC), inhibited intermediate and late gene expression; (**B**) Epoxomicin (Epox), inhibited intermediate and late gene expression and protein degradation by blocking the proteasome.

We investigated Ag expression by two recombinant viruses in which the gene coding for the model Ag ovalbumin (OVA) was under the control of promoters with differential activity at the early and late phases of infection. More specifically, the P7.5 exhibits moderate early and strong late activity, whereas the mPH5 shows a stronger early activity than the P7.5 and similar activity during the late phase of infection [29]. As shown by Western blot analysis, cells infected *in vitro* with both MVA-OVA P7.5 and MVA-OVA mPH5 led to efficient expression of OVA. In contrast to our expectations MVA-OVA mPH5 seemed to express a lower amount of protein than MVA-OVA P7.5 at 2 h post infection (p.i.) under normal conditions (Figure 1A, -AraC). Infected cells were then incubated with proteasomal or viral inhibitors to discriminate if a lower expression or a higher protein turnover was the cause of this finding. Cytosine β-d-arabinofuranoside (AraC) inhibits DNA replication and consequently vaccinia virus intermediate and late gene expression, whereas expression of early viral genes remains unaffected [47]. The results obtained in the presence of AraC clearly show that Ag expression from the mPH5 is stronger than that from P7.5 when only early gene expression is licenced (Figure 1A). The proteasome inhibitor epoxomicin not only abrogates MVA intermediate and late gene expression, while early gene expression remains unaffected, but also prevents proteolytic degradation via the proteasomal pathway, thereby allowing proteins to accumulate [48]. As shown in Figure 1B, the addition of epoxomicin allowed protein accumulation at 2 h p.i. and increased over time, demonstrating that mPH5 indeed has a stronger early activity and OVA expressed under the control of mPH5 is rapidly degraded at an early time point. To rule out any possible artefact due to increased Ag secretion, we analyzed the OVA secreted to the medium. Under normal conditions OVA was not detected in the medium before 4 h p.i. and treatment with brefeldin A, an inhibitor of the secretory pathway, confirmed that OVA started to accumulate in the cells at a late time point (data not shown). Thus, we conclude that there is indeed a rapid turnover of OVA by MVA-OVA mPH5 infected cells at 2 h. These data collectively suggest that the OVA protein expressed under the control of the mPH5 promoter could serve as substrate for Ag processing by proteasomes, therefore increasing the formation of defective ribosomal products (DRiPs), which in turn leads to the apparent faster turnover of the Ag [49,50].

### 3.2. Activation of Murine DCs Is Primarily Dependent on the MOI of rMVA, but It Is Also Affected by the Promoter Selected To Drive Ag Expression

**Figure 2:**
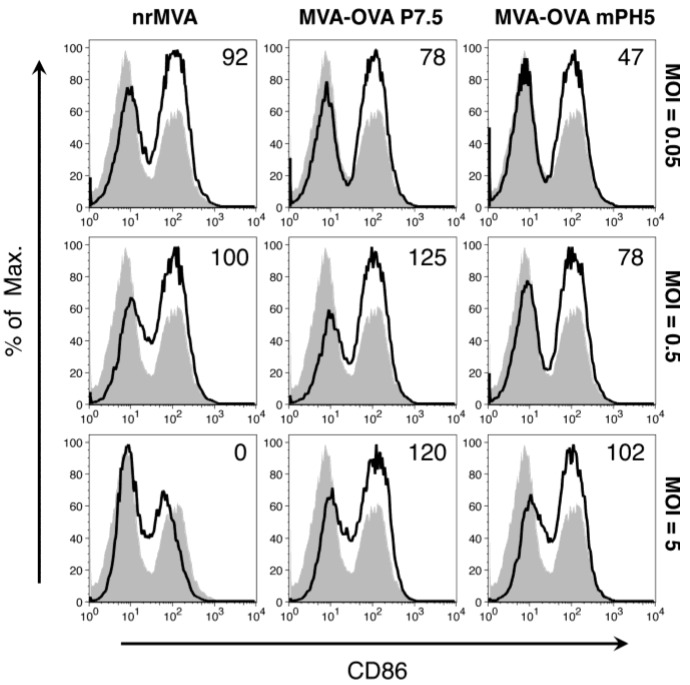
DC CD86 expression depends on the MOI of MVA. DCs were infected with nrMVA, MVA-OVA P7.5, and MVA-OVA mPH5 at different MOIs for 6 h. After washing and further incubation for 16 h, changes in the expression of CD86 were measured by flow cytometry. The MOIs of 0.05, 0.5, and 5 were arbitrarily considered as representative of low, intermediate and high MOI, respectively (open histograms). Mock infected DCs (MVA = 0, shaded histogram) was considered as a basal level of CD86 expression. Numbers in the upper right corners indicate fold-changes as %.

Murine bone marrow (BM)-derived DCs were infected with rMVA at different multiplicities of infection ([MOIs] 0.05, 0.5, and 5), which were in turn considered as low, intermediate and high. The DC activation status was first evaluated by assessing the expression of the co-stimulatory molecules CD80 and CD86. At all tested MOIs, the number of DCs expressing CD86 was higher compared to the non-infected DCs. As we previously reported [30], DCs infected with nrMVA showed a low expression of CD86 at the highest MOI (Figure 2). Cells infected with MVA-OVA P7.5 showed the highest level of CD86 expression at the intermediate MOI and a slightly lower level at the highest MOI. In contrast, DCs infected with MVA-OVA mPH5 showed lower levels of CD86 expression than DCs infected with the other MVAs at the lowest MOI. However, the expression of CD86 increased in a MOI-dependent manner (Figure 2). This suggests that the level of activation of DCs depends not only on the MOI but also on the Ag expression level.

**Figure 3:**
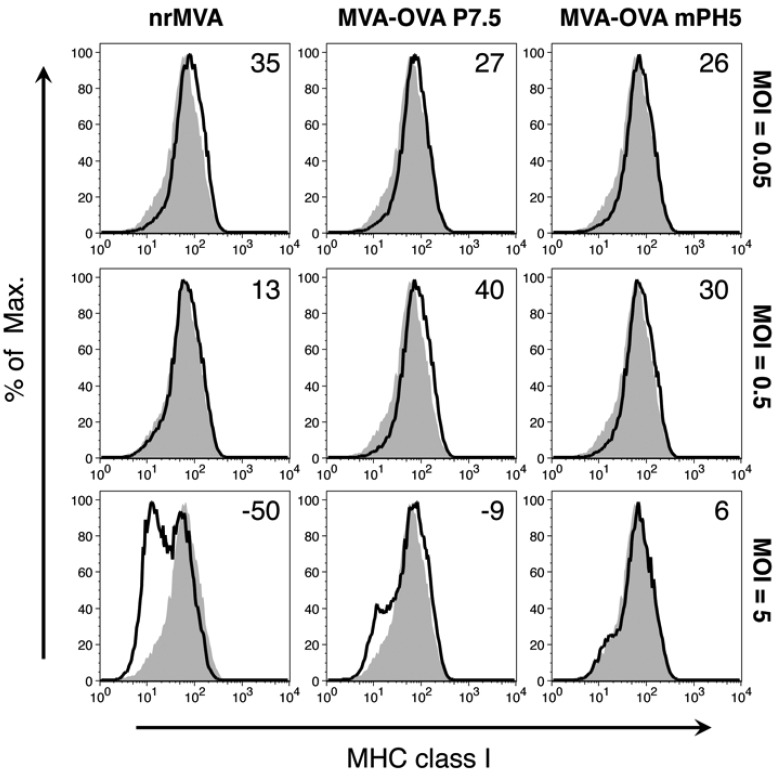
DC expression of MHC class I depends on the MOI of MVA. DCs were infected with nrMVA, MVA-OVA P7.5, and MVA-OVA mPH5 at different MOIs for 6 h. After washing and further incubation for 16 h, changes in the expression of MHC class I were measured by flow cytometry. The MOIs of 0.05, 0.5, and 5 were arbitrarily considered as representative of low, intermediate, and high MOI, respectively (open histograms). Mock infected DCs (MVA = 0, shaded histogram) were considered as a basal level of MHC class I expression. Numbers in the upper right corners indicate fold-changes as %.

It has been previously reported that nrMVA induces down-regulation of MHC class I expression. As expected, DCs infected with nrMVA showed a drastic decrease in the expression of MHC class I in a MOI-dependent manner. However, MHC class I expression by DCs infected with either MVA-OVA P7.5 or MVA-OVA mPH5 was only marginally affected at the highest MOI (Figure 3), particularly in cells infected by MVA-OVA mPH5. The expression of MHC class II (Supplementary Figure S1) followed a decreasing trend similar to MHC class I, whereas expression of CD86 and CD80 showed increasing levels with MOI (Supplementary Figure S2).

Some of these changes may be associated to the cell status and we and others have previously demonstrated that MVA induces cell apoptosis and necrosis at high MOIs in a dose-dependent manner [30]. Thus, we decided to investigate the impact of these rMVAs on cell viability. DC viability after infection with rMVA expressing OVA under the control of the P7.5 and mPH5 promoters was remarkably unaffected (Supplementary Figure S3). How the expression of a foreign Ag by MVA rescues cells from apoptosis is unknown. Although similar observations have been reported in the literature [51], the mechanisms involved are largely unknown and they go beyond the primary scope of this paper.

### 3.3. The Strength of Viral Ag Expression during the Early Phase Shapes Ag Presentation

**Figure 4:**
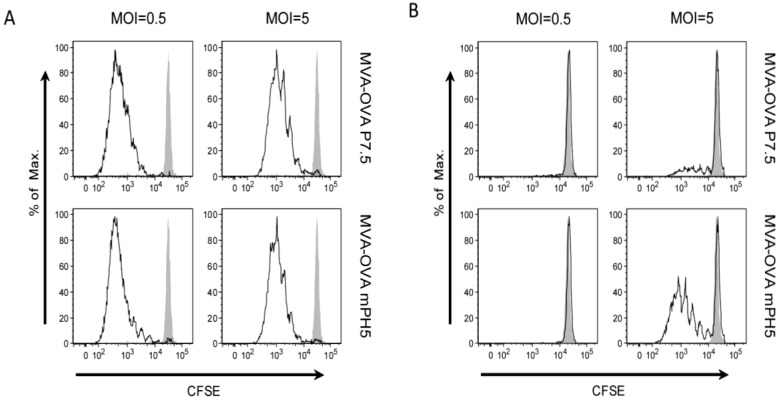
*In vitro* Ag presentation by DCs infected with MVA-OVA. DCs were infected for 6 h with MVA-OVA P7.5 and MVA-OVA mPH5 at a MOI of 0.5 or 5. Then, DCs were co-cultured with either CFSE-labeled CD8^+^ T cells from naïve OT-I mice (**A**) or CFSE-labeled CD4^+^ T cells from naïve OT-II mice (**B**) for 5 days. Proliferation was then assessed by flow cytometry (open histograms). Mock infected DCs were used as control (shaded histograms).

**Figure 5:**
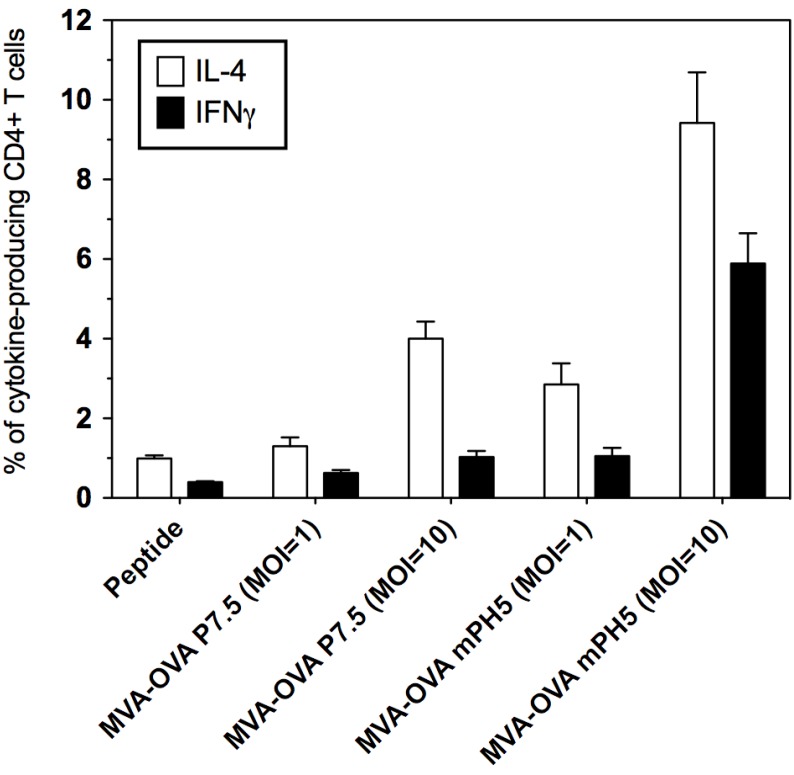
Cytokine production in an *in vitro* Ag presentation test by DCs infected with MVA-OVA. DCs were infected for 6 h with MVA-OVA P7.5 and MVA-OVA mPH5 at a MOI of 1 or 10. After infection, DCs were cultured with naïve CD4^+^ T cells from OT-II mice. At day 5 intracellular staining was carried out for IL-4 and IFN-γ. OVA peptide AA323–339 was used as a control.

The changes observed on activation markers prompted us to evaluate if Ag expression driven by different promoters also affects the Ag presentation capacity of DCs. To this end, Ag presentation by DCs infected with MVA-OVA P7.5 or MVA-OVA mPH5 was evaluated *in vitro*. Ag-specific CFSE-labelled CD8^+^ and CD4^+^ T cells (from OT-I and OT-II mice, respectively) were co-cultured with infected DCs and proliferation was assessed by the dilution of the fluorescent dye (Figure 4). A similarly strong CD8^+^ T cell proliferation was induced by DCs infected with MVA-OVA P7.5 and MVA-OVA mPH5 in a MOI-dependent manner (Figure 4A). However, proliferation of CD4^+^ T cells induced by MVA-OVA mPH5 was stronger than that promoted by MVA-OVA P7.5 (Figure 4B). Furthermore, CD4^+^ T cells stimulated in an Ag presentation assay showed higher levels of IL-4 and particularly IFN-γ production when using MVA-OVA mPH5 infected DCs, as compared to MVA-OVA P7.5 infected DCs (Figure 5).

### 3.4. Immunization with rMVA Expressing OVA Driven by Different Promoters Induced Different Ab Responses

**Figure 6:**
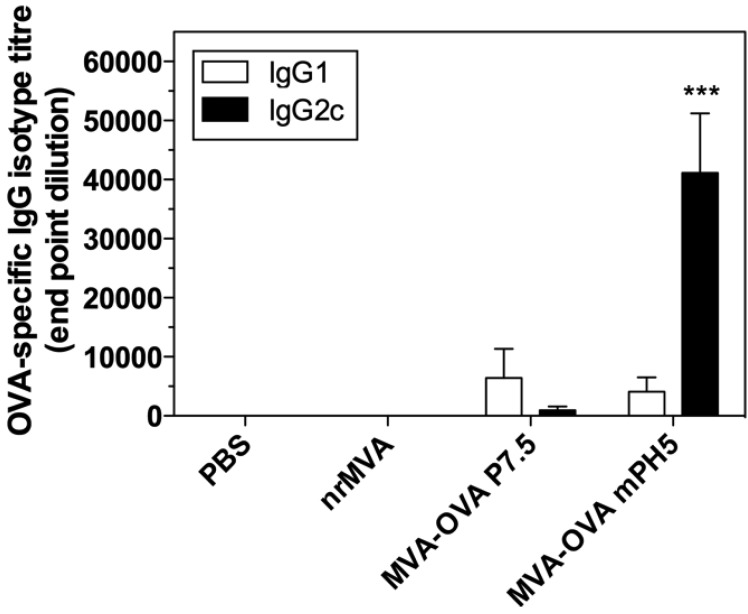
OVA-specific IgG1/IgG2c in sera of mice vaccinated with MVA-OVA P7.5 and MVA-OVA mPH5. OVA-specific IgG1 and IgG2c isotypes were determined by ELISA. Each bar represents the group mean end-point titre, the SEM is indicated by vertical lines. The results were statistically significant at *p* < 0.001 (***) compared to all groups.

*In vitro* results showed that the use of MVA-OVA mPH5 leads to a higher Ag production and induces higher numbers of IFN-γ producing CD4^+^ T cells than MVA-OVA P7.5. Thus, we investigated if these constructs would induce different immune responses in an *in vivo* model. Mice were immunized by i.m. route with MVA-OVA P7.5 or MVA-OVA mPH5 twice (day 0 and 28), and the immune responses elicited were then evaluated. As controls, mice were immunized with either PBS or nrMVA. Immunization with MVA-OVA P7.5 induced only a moderate OVA specific IgG response, whereas immunization with MVA-OVA mPH5 induced a strong OVA-specific IgG response (Figure 6) that is comparable to responses elicited in mice immunized with OVA protein using nrMVA as adjuvant [30]. Conversely to IgG responses, in mice immunized with nrMVA + OVA and MVA-OVA P7.5, MVA-OVA mPH5 IgG responses were characterized by a significantly enhanced production of IgG2c (Figure 6). This in turn indicates stimulation of a dominant Th1 response.

### 3.5. Immunization with rMVA Expressing OVA Driven by Different Promoters Shape the CD8^+^ T Cell Immune Response

**Figure 7:**
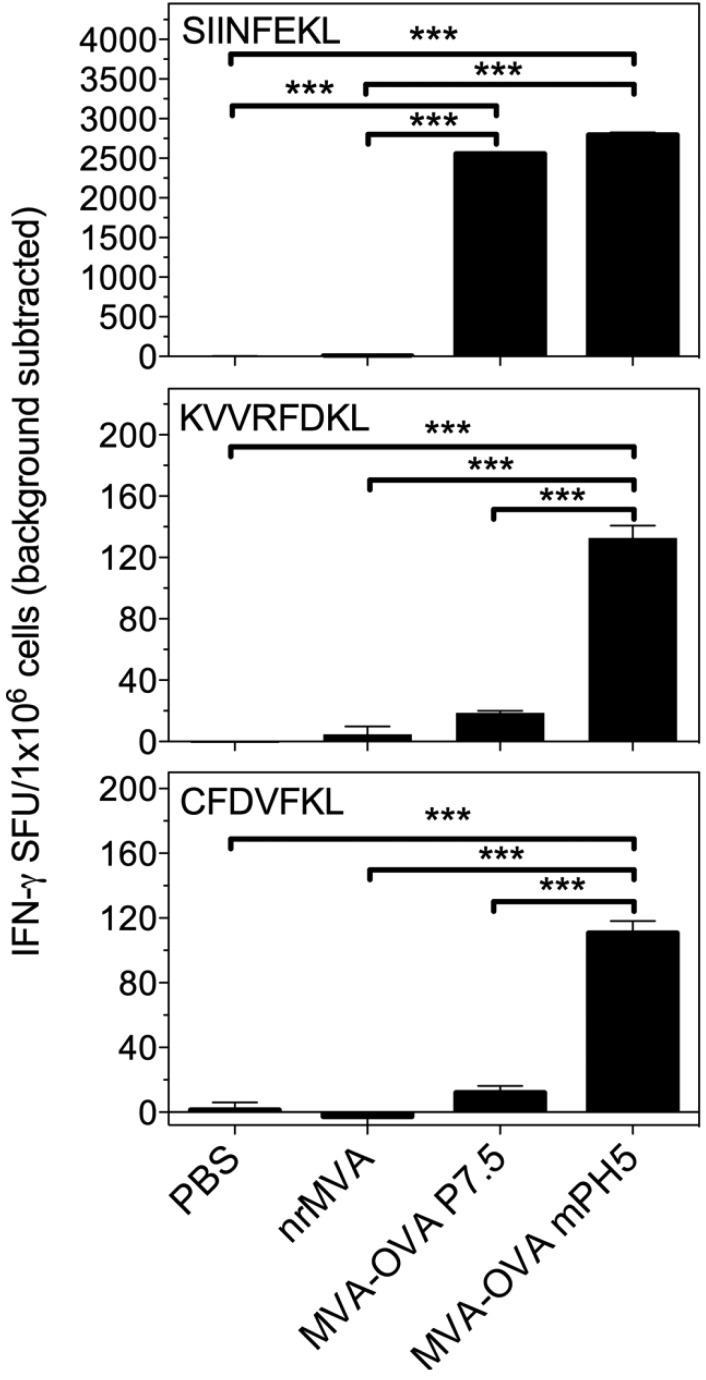
IFN-γ-producing cells in mice vaccinated with MVA-OVA P7.5 and MVA-OVA mPH5. Splenocytes of immunized mice were re-stimulated *in vitro* for 16 h with peptides encompassing the dominant (SIINFEKL) and subdominants (KVVRFDKL and CFDVFKL) MHC class I restricted epitopes of OVA to determine the number of IFN-γ secreting CD8^+^ T cells. Results are presented as specific spot forming units (SFU)/1 × 10^6^ cells. The SEM of quadruplicate values is indicated by vertical lines. The values reported are those obtained from stimulated cells with the background from non-stimulated cells being subtracted. The results were statistically significant at *p* < 0.001 (***).

We then evaluated the induction of Ag-specific CD8^+^ T cells by vaccination with the two rMVA viruses. To this end, we first assessed the number of Ag specific IFN-γ secreting CD8^+^ T cells by intracellular staining when splenocytes were re-stimulated *ex vivo* with a peptide corresponding to the dominant OVA epitope (SIINFEKL). Immunization with both rMVAs expressing OVA resulted in the induction of high numbers of IFN-γ producing CD8^+^ T cells in response to the immune dominant peptide (Supplementary Figure S4). These results were confirmed in an independent experiment by ELISPOT (*p* < 0.001, Figure 7).

Due to the faster turnover of OVA during the early phase in cells infected with MVA-OVA mPH5, we investigated whether CD8 responses to OVA subdominant epitopes were also elicited. Although the number of IFN-γ-producing cells in response to the dominant peptide was similar for both viral vectors, the immune responses elicited to two subdominant epitopes of OVA (KVVRFDKL, CFDVFKEL) were markedly different. Notably, only MVA-OVA mPH5 induced a significant response to both subdominant peptides (Figure 7, *p* < 0.001). This suggests that the fast turnover of the Ag increases the production of a broader range of OVA epitopes that are efficiently presented *in vivo*.

## 4. Discussion

During the last decades, the way to develop new vaccines has radically changed. A more rational vaccine design has been fostered by major advances in immunology and vaccinology. The better understanding of Ag presentation pathways for MHC class I restricted epitopes and improved exploitation of viral vectors has led to the development of strategies to stimulate strong CD8^+^ T cell responses. MVA is one of the most studied and exploited viral vectors. This vector is of particular interest because of its excellent safety record when administered during the smallpox eradication campaign [6,7,8] and its capacity to accommodate large inserts [38,52]. Furthermore, many of the genes involved in immune escape were lost during its attenuation, thereby proving an excellent starting point for the generation of new vaccines [10,11,12,13,14,15,16]. In addition, recent studies by us and others have unravelled some of the mechanisms involved in Ag presentation by MVA infected cells, thereby providing the knowledge base for generating better constructs [25,28,53,54,55,56].

It is known that endogenously produced Ags can be directly or cross-presented to CD8^+^ T cells. Considering the fact that DCs are professional APCs and that MVA infected DCs exhibit strong Ag expression and presentation, both *in vitro* and *in vivo*, a role in direct priming of CD8^+^ T cells seems initially the most likely pathway. However, we have previously demonstrated that vaccination of mice with MVA encoding an unstable Ag was not able to prime strong CD8^+^ T cell responses [25]. We also showed that the primary CD8^+^ T cell response induced by MVA vaccination is mainly induced by DCs that acquire Ag from other infected cells and cross-present it to naïve T cells [25]. Thus, the most relevant pathway to induce CD8^+^ T cell responses *in vivo* is cross-priming. In this work, we compared the immune response evoked by DCs infected with MVA-OVA P7.5 and MVA-OVA mPH5. The main difference between these two vaccine vectors relies on the pattern of Ag expression during the early phase. The mPH5 is a stronger promoter than P7.5, and our data show that the over expression driven by the mPH5 promoter leads to a higher Ag production which is rapidly degraded. However, it also leads to long-lived Ags suitable for cross-presentation, presumably produced at later stages of infection. Mice vaccinated with MVA-OVA mPH5 showed a similar CD8^+^ T cell response to the immune dominant OVA peptide as compared to mice vaccinated with MVA-OVA P7.5. However, animals vaccinated MVA-OVA mPH5 also showed a significantly higher response to the subdominant epitopes of OVA. Due to the fact that degraded products are usually not good substrates for cross-presentation, a plausible explanation is that a small proportion of cells expressing the Ag during the early infection phase are able to directly present the rapidly degraded protein. Thus, although cross-presentation still remains the main pathway of presentation, these new data highlight the importance of Ag expression during the early phase of infection in shaping the immune dominance hierarchy [28]. It is important to mention that in contrast to previous studies where MVA-infected cells expressed an ubiquitylated Ag, which was completely degraded, the fast turnover induced by overexpression driven by the mPH5 mainly occurs during the early infection phase. Thus, MVA-infected cells not only have the degraded products, but also long-lived Ag required for cross-presentation and effective boosting. A recent work by Yewdell’s group suggested that MHC class I Ag processing distinguishes endogenous Ags, based on their translation from cellular versus viral mRNA [57]. It is possible then, although this is only speculation, that the MHC I Ag processing is enhanced when the gene is under the control of a strong viral promoter in the early phase of infection compared to those intermediate and late genes that are active only during viral DNA replication.

Interestingly, mice vaccinated with MVA-OVA mPH5 produce high levels of OVA-specific IgG2c and strong IFN-γ responses by CD4 cells *in vivo*. Furthermore, in an *in vitro* Ag-presentation assay, low levels of IL-4 production were detected. These results indicate that the strength of the promoter during the early phase and the stability of the Ag influence the T helper profile, leading in the case of mPH5 to a strong Th1 pattern.

## 5. Conclusions

We showed that overexpression driven by a strong promoter during the early phase, like mPH5, results in the production of Ags that are rapidly degraded by the proteasome, as well as long-lived Ags suitable for cross-presentation. In contrast to cells infected with MVA vectors in which promoters that direct production of either unstable Ags that are fully degraded or long-lived Ags for cross-presentation, the MVA carrying the mPH5 promoter has the capability of doing both. This results in a broader repertoire of T cell specificities and maintenance of the priming efficiency of stable Ags. In addition, the high levels of Ag and the robust CD4 T helper response support enhanced Ab responses. Thus, the mPH5 promoter is a new tool to be considered for fine-tuning responses elicited by vaccines based on rMVA and related vectors.

## Supplementary Files

Supplementary File 1
