# Supplementary material for: Gene Expression Driven by a Strong Viral Promoter in MVA Increases Vaccination Efficiency by Enhancing Antibody Responses and Unmasking CD8+ T Cell Epitope
Source: Vaccines (Basel). 2014 Jul 22;2(3):581–600. doi: 10.3390/vaccines2030581 (PMC4494220; doi:10.3390/vaccines2030581)

**Supplementary Material for**

Gene Expression Driven by a Strong Viral Promoter in MVA Increases Vaccination Efficiency by Enhancing Antibody Responses and Unmasking CD8^+^ T Cell Epitopes

**Material and Methods**

***Detection of Ag Specific Abs***

In brief, 96-well Immuno MaxiSorp plates (Nunc, Roskilde, Denmark) were coated overnight at
4 °C with 100 µL of OVA with a concentration of 5 µg/mL in carbonated buffer (pH 9.6). Then, the wells were blocked and the plates were washed and further incubated with 100 µL of serial two-fold dilutions of sera at 37 °C for 1 h. After four washes, the biotinylated γ-chain specific goat anti-mouse detection Ab (Sigma Chemie, Deisenhofen, Germany) was added. The plates were further incubated
at 37 °C for 1 h. After the plates were washed, HRP-conjugated streptavidin (PharMingen, San Diego, CA, USA) was added and the plates were incubated at 37 °C for 45 min. After another four washes, the reaction was developed using ABTS in 0.1 M citrate-phosphate buffer (pH 4.35) containing 0.01% H_2_O_2_, and the absorbance was read at a wavelength of 405 nm. The IgG isotypes present in the serum samples were determined by ELISA using as secondary Abs biotin-conjugated rat anti-mouse IgG1 and IgG2c (Southern Biotechnology Associates, Birmingham, AL). The absorbance values were plotted against the dilutions, and the endpoint titres were determined as the highest sample dilution that gave an A_405_ of ≥0.2 above the background values. The results were expressed as mean ± SEM for each group.

***Detection of IFN-γ-Producing Cells by ELISPOT***

The numbers of IFN-γ-secreting cells in the spleen of immunized mice were determined by ELISPOT assay, according to the manufacturer’s instructions (BD Bioscience, San Diego, CA, USA). Briefly, polyvinylidene difluoride plates (Millipore, Bedford, MA, USA) were coated with anti-IFN-γ specific capture Abs (100 µL/well). After washing and blocking, cells (5 × 10^5^ cells/well) were incubated in triplicate in the absence or presence of MHC class I restricted OVA peptide for 16 h. Then, plates were washed and 100 µL of the corresponding biotinylated detection Ab was added to each well, and the plates were incubated for 1 h at room temperature. After several washes, the plates were further incubated for 1 h with 100 µL/well of HRP-conjugated streptavidin (BD Bioscience). The spots were developed by using 3-amino-9-ethylcarbazole (Sigma) in 0.1 M acetate buffer (pH 5.0) and 0.05% H_2_O_2_. The reaction was stopped by rinsing the plates with tap water, and the plates were air-dried. The spots were scanned with a CTL ELISPOT reader (ImmunoSpot series 3A) and were counted by using ImmunoSpot image analyzer software v3.2.

**Figure S1.** DC expression of MHC class II depends on the MOI of MVA. DCs were infected with nrMVA, MVA-OVA P7.5 and MVA-OVA mPH5 at different MOIs for 6 h. After washing and further incubation for 16 h, changes in the expression of MHC class II were measured by flow cytometry. The MOIs of 0.05, 0.5 and 5 were arbitrarily considered as representative of low, intermediate and high MOI, respectively (open histograms). Mock infected DCs (MVA = 0, shaded histogram) were considered as a basal level of MHC class II expression. Numbers in the upper right corners indicate fold-changes as %.


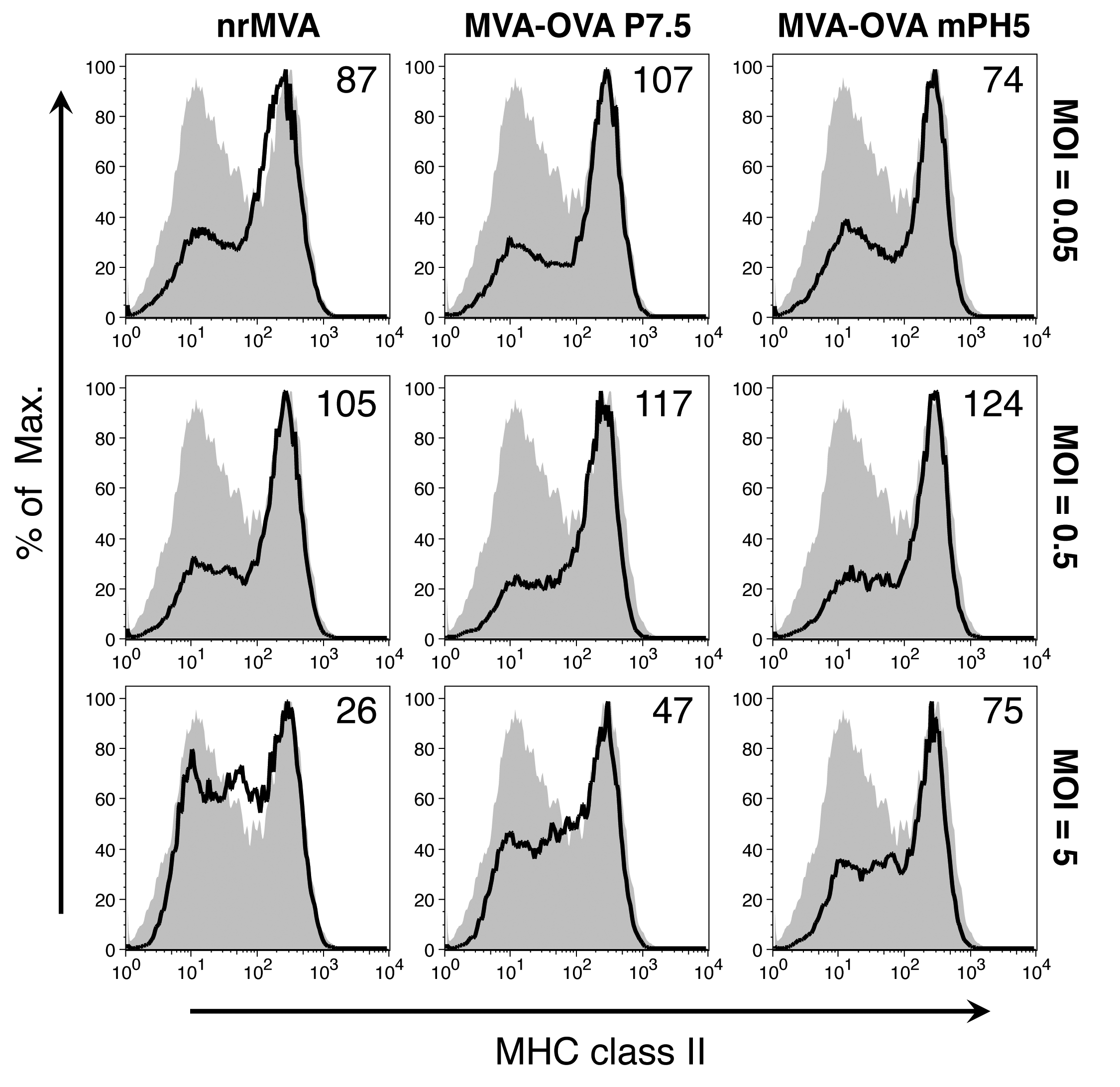


**Figure S2.** DC CD80 expression depends on the MOI of MVA. DCs were infected with nrMVA, MVA-OVA P7.5 and MVA-OVA mPH5 at different MOIs for 6 h. After washing and further incubation for 16 h, changes in the expression of CD80 were measured by flow cytometry. The MOIs of 0.05, 0.5 and 5 were arbitrarily considered as representative of low, intermediate and high MOI, respectively (open histograms). Mock infected DCs (MVA = 0, shaded histogram) were considered as a basal level of CD80 expression. Numbers in the upper right corners indicate fold-changes as %.

OT-I

**A**

OT-II


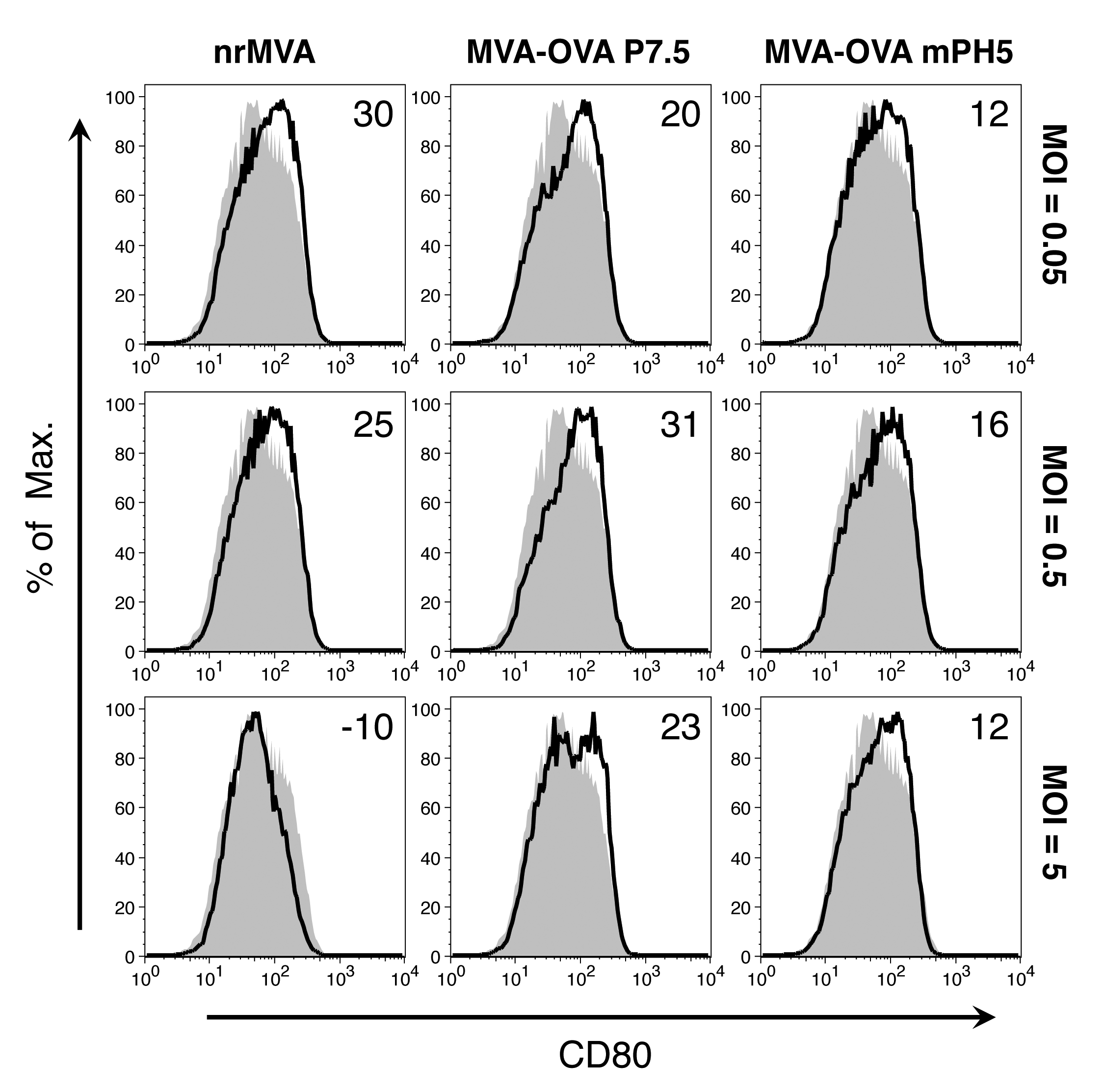


**Figure S3.** DC viability depends on the MOI of MVA. DCs were infected with nrMVA, MVA-OVA P7.5 and MVA-OVA mPH5 at different MOIs for 6 h. After washing and further incubation for 16 h, DC viability was assessed by FACS using V-Annexin (apoptotic cells) and 7-AAD (necrotic cells). The MOIs of 0.05, 0.5 and 5 were arbitrarily considered as representative of low, intermediate and high MOI, respectively.

OT-I

**A**

OT-II


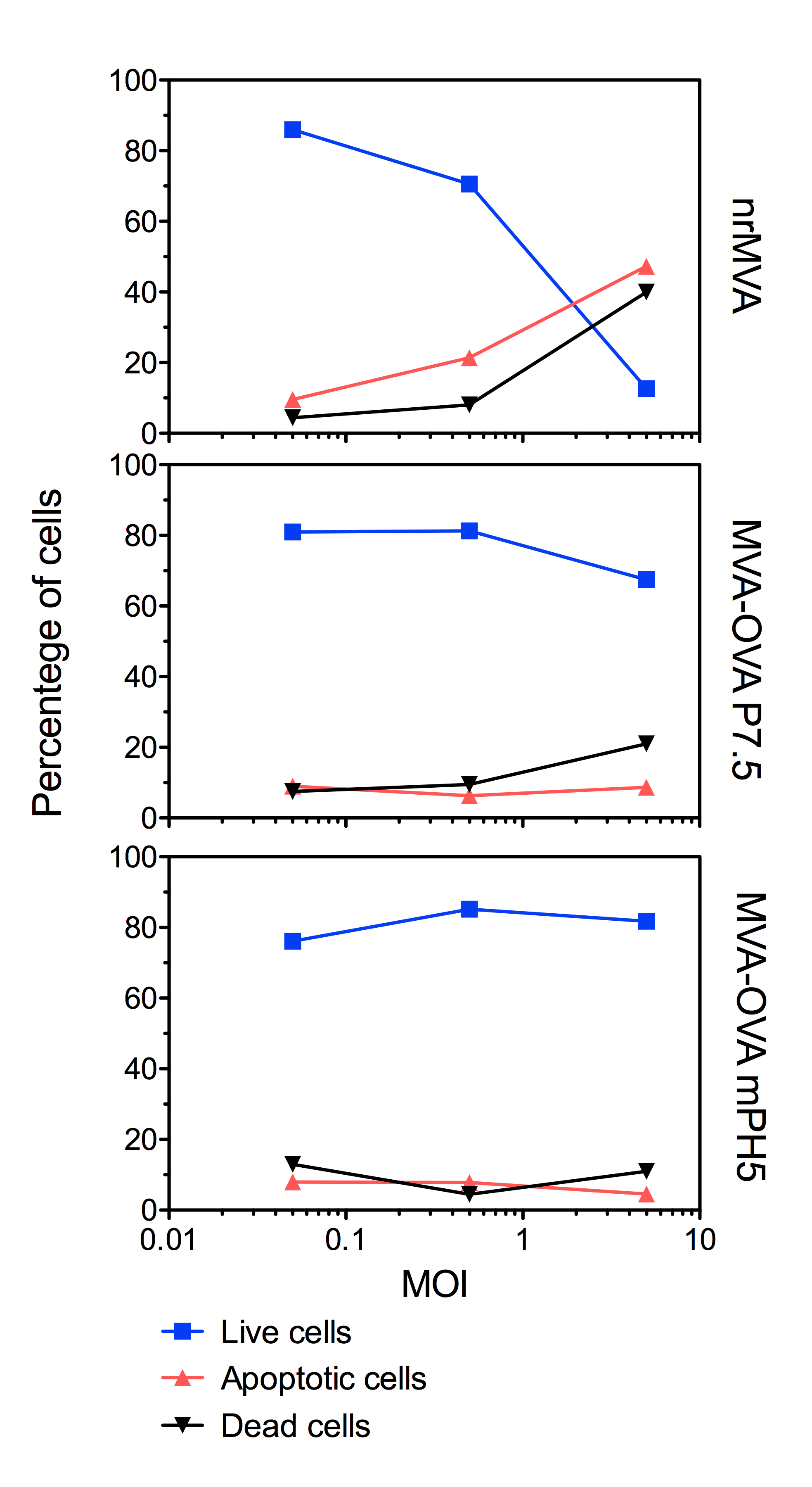


**Figure S4.** IFN-γ-producing cells in mice vaccinated with MVA-OVA P7.5 and
MVA-OVA mPH5. Splenocytes of immunized mice were re-stimulated *in vitro* with peptides encompassing the dominant MHC class I restricted epitopes of OVA (SIINFEKL) an analysed by intracellular staining as described in material and methods section. The number of IFN-γ producing cells is expressed as a % of CD8^+^ T cells.


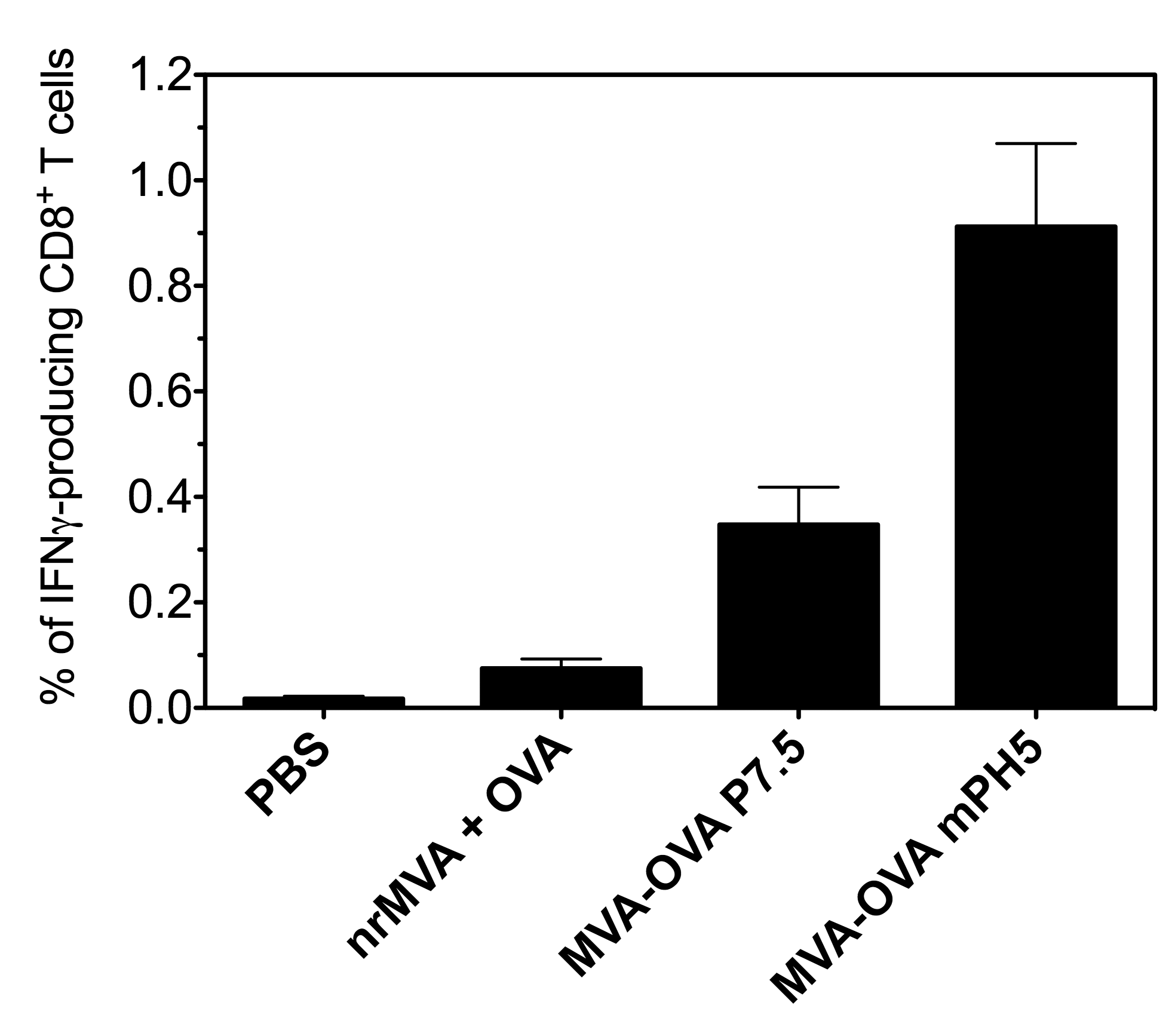

Supplement: Supplementary File 1 [file vaccines-02-00581-s001.doc]
